# Asymmetry indicates more severe and active disease in Graves’ orbitopathy: results from a prospective cross-sectional multicentre study

**DOI:** 10.1007/s40618-020-01258-w

**Published:** 2020-05-30

**Authors:** P. Perros, M. P. Žarković, G. C. Panagiotou, C. Azzolini, G. Ayvaz, L. Baldeschi, L. Bartalena, A. M. Boschi, M. Nardi, T. H. Brix, D. Covelli, C. Daumerie, A. K. Eckstein, N. Fichter, S. Ćirić, L. Hegedüs, G. J. Kahaly, O. Konuk, J. J. Lareida, O. E. Okosieme, M. Leo, L. Mathiopoulou, L. Clarke, F. Menconi, D. S. Morris, J. Orgiazzi, S. Pitz, M. Salvi, I. Muller, M. Knežević, W. M. Wiersinga, N. Currò, C. M. Dayan, C. Marcocci, M. Marinò, L. Möller, S. H. Pearce, F. Törüner, M. Bernard, The European Group on Graves’ Orbitopathy, P. Perros, M. P. Žarković, G. C. Panagiotou, C. Azzolini, G. Ayvaz, L. Baldeschi, L. Bartalena, A. M. Boschi, M. Nardi, T. H. Brix, D. Covelli, C. Daumerie, A. K. Eckstein, N. Fichter, S. Ćirić, L. Hegedüs, G. J. Kahaly, O. Konuk, J. J Lareida, O. E. Okosieme, M. Leo, L. Mathiopoulou, L. Clarke, F. Menconi, D. S. Morris, J. Orgiazzi, S. Pitz, M. Salvi, I. Muller, M. Knežević, W. M. Wiersinga, N. Currò, C. M. Dayan, C. Marcocci, M. Marinò, L. Möller, S. H. Pearce, F. Törüner, M. Bernard

**Affiliations:** 1 Department of Endocrinology, Level 6, Leazes Wing, Royal Victoria Infirmary, Newcastle upon Tyne, NE1 4LP Tyne UK; 2 Faculty of Medicine, University of Belgrade, Dr Subotića 8, Belgrade, Serbia; 3 Department of Medicine and Surgery, Section of Ophthalmology, School of Medicine, University of Insubria, Via Guicciardini 9, 21100 Varese, Italy; 4 Department of Endocrinology, Yüksek Ihtisas University Ankara Koru Hastanesi, 1450. Sk. No:13, Kızılırmak, 06510 Çankaya, Ankara Turkey; 5 Department of Ophthalmology, Université Catholique de Louvain, Cliniques Universitaires Saint-Luc, Brussels, Belgium; 6 Endocrine Unit, University of Insubria, Ospedale di Circolo, Viale Borri, 57 21100 Varese, Italy; 7 Dipartimento di Patologia Chirurgica Medica, Molecolare e Dell’Area Critica, Università di Pisa, Pisa, Italy; 8 Department of Endocrinology and Metabolism, Odense University Hospital, 5000 Odense, Denmark; 9 Graves’ Orbitopathy Center, Endocrinology, Fondazione IRCCS Cà Granda, University of Milan, via Sforza, 35 - I-20122, Milan, Italy; 10 Department of Endocrinology, Université Catholique de Louvain, Cliniques Universitaires Saint-Luc, Brussels, Belgium; 11 Department of Ophthalmology, University of Duisburg-Essen, 45122 Essen, Germany; 12 Interdisciplinary Centre for Graves’ Orbitopathy, 4600 Olten, Switzerland; 13 Clinic of Endocrinology, Clinical Centre of Serbia, Belgrade, Serbia; 14 Department of Medicine I, Johannes Gutenberg University Medical Center, 55101 Mainz, Germany; 15 Department of Ophthalmology, Faculty of Medicine, Gazi University, Besevler, Ankara, 06500 Turkey; 16 Thyroid Research Group, Cardiff University School of Medicine, Cardiff, UK; 17 Department of Clinical and Experimental Medicine, University of Pisa, Via Paradisa 2, 56124 Pisa, Italy; 18 Newcastle Eye Centre, Royal Victoria Infirmary, Newcastle upon Tyne, UK; 19 Cardiff Eye Unit, University Hospital of Wales, Cardiff, UK; 20 Department of Endocrinology, Centre Hospitalier Lyon-Sud, Lyon, France; 21 Orbital Center, Ophthalmic Clinic, Bürger Hospital, Frankfurt, Germany; 22 Medical School, Clinic for Ophthalmology, Clinical Center of Serbia, University of Belgrade, Belgrade, Serbia; 23 Department of Endocrinology, Academic Medical Center, Amsterdam, Netherlands; 24 Department of Surgery, Ophthalmology Unit, Fondazione IRCCS Ca’ Granda Ospedale Maggiore Policlinico, Milan, Italy; 25 Department of Endocrinology, Faculty of Medicine, Gazi University, Besevler, Ankara, 06500 Turkey; 26 Neuro-Ophthalmology Outpatient Clinics, GHE-Hospices Civils de Lyon and Lyon 1 University, Lyon, France

**Keywords:** Graves’, Orbitopathy, Thyroid, Asymmetric, Unilateral

## Abstract

**Purpose:**

Patients with Graves’ orbitopathy can present with asymmetric disease. The aim of this study was to identify clinical characteristics that distinguish asymmetric from unilateral and symmetric Graves’ orbitopathy.

**Methods:**

This was a multi-centre study of new referrals to 13 European Group on Graves’ Orbitopathy (EUGOGO) tertiary centres. New patients presenting over a 4 month period with a diagnosis of Graves’ orbitopathy were included. Patient demographics were collected and a clinical examination was performed based on a previously published protocol. Patients were categorized as having asymmetric, symmetric, and unilateral Graves’ orbitopathy. The distribution of clinical characteristics among the three groups was documented.

**Results:**

The asymmetric group (*n* = 83), was older than the symmetric (*n* = 157) group [mean age 50.9 years (SD 13.9) vs 45.8 (SD 13.5), *p* = 0.019], had a lower female to male ratio than the symmetric and unilateral (*n* = 29) groups (1.6 vs 5.0 vs 8.7, *p* < 0.001), had more active disease than the symmetric and unilateral groups [mean linical Activity Score 3.0 (SD 1.6) vs 1.7 (SD 1.7), *p* < 0.001 vs 1.3 (SD 1.4), *p* < 0.001] and significantly more severe disease than the symmetric and unilateral groups, as measured by the Total Eye Score [mean 8.8 (SD 6.6) vs 5.3 (SD 4.4), *p* < 0.001, vs 2.7 (SD 2.1), *p* < 0.001].

**Conclusion:**

Older age, lower female to male ratio, more severe, and more active disease cluster around asymmetric Graves’ orbitopathy. Asymmetry appears to be a marker of more severe and more active disease than other presentations. This simple clinical parameter present at first presentation to tertiary centres may be valuable to clinicians who manage such patients.

## Introduction

Graves’ orbitopathy (GO) is an orbital disorder closely associated with autoimmune thyroid disease [1]. GO is responsible for considerable psychosocial morbidity, personal and societal cost [2, 3], can rarely cause blindness [4], and is associated with increased mortality including that from suicide [5, 6]. Most patients present with bilateral disease though asymmetric or unilateral disease is also observed in a minority of cases. The prevalence of unilateral disease is 4.5–14% [7–11], while asymmetric disease is reported to occur in 9–34% of patients with GO [9–12]. Although the underlying pathogenesis of all forms of GO is likely to be the same and based on autoimmunity, asymmetric and unilateral GO may differ in the relative contributions of mechanical and inflammatory influences as well as local vascular or anatomical factors. The objective of this study was to analyze data from a prospective cross-sectional multi-centre study [13] and identify clinical characteristics that distinguish asymmetric, unilateral and bilateral symmetric GO.

## Methods

### Patients

All new referrals of patients with a diagnosis of GO to EUGOGO centres were recruited prospectively during a 4-month period between 1st September 2012 and 31st December 2012. Data were collected during their first consultation as described in Perros et al. [13].

### Assessments

Ophthalmic signs were assessed according to established EUGOGO protocols [13]. All assessors had received specialist training in the clinical evaluation of patients with GO and had attended at least one EUGOGO teaching course. Detailed description of assessments can be found in Perros et al. [13]. Unilateral disease was defined as one or more clinical features of GO in one eye without any evidence of GO in the contralateral eye. Asymmetric GO was defined as disease present in both eyes with one or more of the following features: difference between the two eyes in exophthalmometer readings by two or more mm; difference in palpebral aperture by two or more millimeter, difference in eyelid swelling by one or more grades; difference in eyelid erythema by one or more grades; difference in conjunctival redness by one or more grades; presence of dysthyroid optic neuropathy in one eye only. Duration of GO was ascertained based on the patient’s history. This study was observational to aid audit and service development by EUGOGO centres, and ethical committee approval was deemed not to be necessary [13]. Confidentiality was preserved by pseudoanonymizing the data (pseudonymization substitutes the identity of the data subject in such a way that additional information is required to re-identify the data subject). The described research adhered to the tenets of the Declaration of Helsinki.

### Statistical analyses

To compare clinical characteristics, group linear model ANOVA was used [14]. Pearson Chi-square tests or Fisher’s exact test and trend test for ordinal variables were used where appropriate. The software “R” was used for statistical analysis.

## Results

There were 269 patients with a diagnosis of GO of whom 157 had symmetric (58.4%), 83 asymmetric (30.9%) and 29 (10.7%) unilateral disease. Although there was an overall slight excess of higher right sided exophthalmometer readings, the differences in distribution of exophthalmos measurements between right and left for the entire group, within and between groups, were not statistically significant. The baseline characteristics of patients are shown in Table 1. Comparisons between groups showed that the asymmetric group had an older age than the symmetric group [mean age 50.9 years (SD 13.9) vs 45.8 (SD 13.5), *p* = 0.019], lower female to male ratio than the other two groups (1.6 vs 5.0 vs 8.7, *p* < 0.001), had more active disease than the symmetric and unilateral groups [mean Clinical Activity Score 3.0 (SD 1.6) vs 1.7 (SD 1.7), p < 0.001 vs 1.3 (SD 1.4), *p* < 0.001] and significantly more severe disease than the symmetric and unilateral groups, as measured by the Total Eye Score [mean 8.8 (SD 6.6) vs 5.3 (SD 4.4), *p* < 0.001, vs 2.7 (SD 2.1), *p* < 0.001]. The unilateral group had less severe disease than either of the other two groups (*p* < 0.001) (Table 2). There were no significant differences in all other clinical characteristics (country of origin, type of underlying thyroid disease, type of treatment for thyroid disease, time since diagnosis of thyroid disease, duration of GO, coexistent diabetes, family history of thyroid disease, family history of autoimmune disease, smoking). Thus, the asymmetric group emerged as the most distinct compared with the others, with an older mean age, lowest female to male ratio, more active and more severe GO. Pooling the asymmetric and unilateral groups together and comparing them with the symmetric group did not alter the above findings.

**Table 1:** Clinical characteristics of patients

	Symmetric	Asymmetric	Unilateral
% (No.)	58.4 (157)	30.9 (83)	10.8 (29)
Age [years, mean (SD)]	45.8 (13.5)	50.9 (13.9)	48.6 (15.2)
Female [% (No.)]	83.4 (130)	61.4 (74)	89.7 (26)
Nation [% (No.)]			
Belgium	3.8 (6)	7.2 (6)	3.4 (1)
Denmark	2.5 (4)	0 (0)	6.9 (2)
France	10.8 (17)	16.9 (14)	20.7 (6)
Germany	24.2 (38)	27.7 (23)	27.6 (8)
Italy	30.6 (48)	31.3 (26)	13.8 (4)
Serbia	6.4 (10)	8.4 (7)	0 (0)
Switzerland	7 (11)	1.2 (1)	6.9 (2)
Turkey	5.1 (8)	3.6 (3)	13.8 (4)
UK	9.6 (15)	3.6 (3)	6.9 (2)
Previous thyroid treatment [% (No.)]			
Radioiodine	17.2 (27)	15.7 (13)	24.1 (7)
Thyroidectomy	22.3 (35)	22.9 (19)	17.2 (5)
Anti-thyroid drugs	43.9 (69)	43.4 (36)	41.4 (12)
No treatment	16.6 (26)	18.1 (15)	17.2 (5)
CAS [mean (SD)]^a^	1.7 (1.7)	3.0 (1.6)	1.3 (1.4)
TES [mean (SD)]^a,b^	5.3 (4.4)	8.8 (6.6)	2.7 (2.2)
Difference in exophthalmos between right and left eye^a^ [mm, mean (SD)]	0.5 (5)	2.5 (1.6)	2.2 (2.2)
Difference in PA between right and left eye^a^ [mm, mean (SD)]	1.1 (1.4)	1.7 (1.6)	2.1 (1.6)
Current smoker (No. %)	62 (39.5)	29 (39.8)	13 (44.8)
Duration of GO^a^ (months)	27.8 (46.1)	43.2 (98.7)	22.7 (38.8)

*CAS* clinical activity score, *TES* total eye score, *PA* palpebral aperture, *GO* Graves’ orbitopathy

^a^Mean (standard deviation); ^b^worse TES of the two eyes

**Table 2:** Statistically significant differences between the three groups

	Symmetric	Asymmetric	Unilateral
No. (%)	157 (58.4)	83 (30.9)	29 (10.8)
Age [years, mean (SD)]^a^	45.8 (13.5)	50.9 (13.9)	48.6 (15.2)
Female (No. %)^b^	130 (83.4)	74 (61.4)	26 (89.7)
CAS [mean (SD)]^c^	1.7 (1.7)	3.0 (1.6)	1.3 (1.4)
TES [mean (SD)]^d,e^	5.3 (4.4)	8.8 (6.6)	2.7 (2.2)

*CAS* clinical activity score, *TES* total eye score

^a^asymmetric vs symmetric *p* = 0.019; ^b^asymmetric vs symmetric and asymmetric vs unilateral, *p* < 0.001; ^c^asymmetric vs symmetric and asymmetric vs unilateral, *p* < 0.001; ^d^asymmetric vs symmetric and asymmetric vs unilateral, *p* < 0.001; ^e^unilateral vs symmetric, *p* < 0.001

## Discussion

Facial asymmetry is important as it can be a cause of distress to patients [15], including a negative impact on the quality of life in patients with asymmetric GO [16]. Asymmetry is also an intriguing observation in diseases that are thought to be systemic, and further study may provide insights into factors that modulate disease expression. Asymmetrical or unilateral GO may precede bilateral disease in some patients [11, 17], a fact that is of importance in timing of interventions such as rehabilitative surgery. Asymmetry in the anatomy of the bony orbit, optic canal, ophthalmic vessels, length of the globe, and exophthalmometer readings of normal subjects is common [18–20], but whether structural variations in the orbits are more pronounced in patients with asymmetric/unilateral GO is unknown. The prevalence of asymmetric disease in our cohort was 30.9% and that of unilateral disease 10.7%, which is comparable to reports in published literature of asymmetric in 9–34% [9, 10, 12, 16, 17, 22] and unilateral disease in 4.5–14.0% of patients with GO [7–11]. The variations in the above figures between studies are probably due to differences in definitions of asymmetry and unilaterality used, and inter-/intra-observer variation. In addition, differences in the phase of the natural history of GO at the time of assessment may also have contributed, as after the passage of time some patients with unilateral disease develop bilateral features [11, 17]. An association between asymmetric disease and older age [22, 23], more active disease [22], and lower female to male ratio [24], has been previously reported, and our study has confirmed these associations. The clustering of all the above features as well as increased severity around asymmetric GO is a finding that has not been described before. The reason why asymmetry should be associated with older age and a lower female to male ratio is unclear, though an association between older age and male gender with more severe disease has been previously observed [23–25] and may be partly explained by differences in exposure to smoking. The data from this study however, suggest that smoking is an unlikely explanation for this cohort as the rates of smoking were very similar between the asymmetric and symmetric groups and highest in the unilateral group, though the differences were not statistically significant (Table 1). This study also showed that patients with unilateral disease have significantly milder GO than symmetric or asymmetric variants. Possible explanations for this observation include the fact that patients with unilateral mild GO are more likely to be referred to tertiary centres than bilateral or asymmetric GO of similar severity, for exclusion of other diagnoses, in accordance with published guidelines [26], as well as the association of unilateral disease with euthyroidism and low TSH receptor antibodies [27]. The explanation for why asymmetric/unilateral disease occurs, may partly lie in underlying asymmetry of the bony orbit, which is a normal finding in all people [19] as discussed above, however additional contributors are likely to play a role. A previous study tested the hypothesis that sleeping position may be responsible, but no such association was found [12]. It is possible that differences in vascularity between orbits may be at play, although this has not been studied specifically in asymmetric disease. However, differences in arterial and venous flow in orbital vessels in patients with and without GO have been described, as well as in patients with and without dysthyroid optic neuropathy [28]. Other local factors relating to anatomy and properties of soft tissues (e.g. of the elasticity of the orbital septae), asymmetrical distribution of resident inflammatory cells in the orbit and molecular differences in potential for adipogenesis and production of proinflammatory cytokines by orbital tissues, and unilateral triggers such as infection, may also contribute. The natural history of asymmetric and unilateral disease is not well studied, but the available evidence suggests that in a significant proportion of patients with unilateral disease (14–36%), bilaterality eventually ensues in due course [11,17], so the natural course of the disease in one orbit may lag behind the other. The strengths of our study were the large number of patients included, its multi-centre and multi-national design, clinical assessments based on well-defined protocols, and prospective recruitment over a 4 month period simultaneously by all centres. Weaknesses included absence of detailed motility data (other than Gorman score), potential errors due to multiple observer variation, lack of imaging studies and absence of longitudinal follow-up data, which is crucial in understanding how these subtypes of GO inter-relate, progress from one to another and, most importantly, how do they respond to therapy. The management of GO can be difficult and it is recommended that all but the mildest of cases are referred to tertiary centres [26]. It is not known whether early intervention diminishes the risk or incidence of asymmetric disease, or whether the ultimate prognosis is worse than other phenotypes in terms of disease severity, and associated psycho-social burden, although a negative association with quality of life has been reported [16]. Nonetheless, medical treatments are most effective during the active phase of the disease and recognizing and focusing resources during this window is important. Our data suggest that asymmetry may be a marker of greater disease activity and severity, which can be a useful indicator for prioritizing cases when referrals are received by tertiary centres. In conclusion, this study has shown that asymmetric GO is associated with older age, a lower female to male ratio, more active and more severe disease than symmetric or unilateral GO. Clinicians who manage patients with GO should be aware that patients with asymmetric GO have more active and more severe disease.

## Data Availability

From corresponding author.
